# Musical expertise shapes visual-melodic memory integration

**DOI:** 10.3389/fpsyg.2022.973164

**Published:** 2022-10-24

**Authors:** Martina Hoffmann, Alexander Schmidt, Christoph J. Ploner

**Affiliations:** ^1^Berlin Center for Musicians’ Medicine, Charité – Universitätsmedizin Berlin, Berlin, Germany; ^2^Hanns Eisler School of Music Berlin, Kurt-Singer-Institute for Music Physiology and Musicians’ Health, Berlin, Germany; ^3^Department of Neurology, Charité – Universitätsmedizin Berlin, Berlin, Germany

**Keywords:** musical memory, visual memory, memory integration, musicianship, associative inference

## Abstract

Music can act as a mnemonic device that can elicit multiple memories. How musical and non-musical information integrate into complex cross-modal memory representations has however rarely been investigated. Here, we studied the ability of human subjects to associate visual objects with melodies. Musical laypersons and professional musicians performed an associative inference task that tested the ability to form and memorize paired associations between objects and melodies (“direct trials”) and to integrate these pairs into more complex representations where melodies are linked with two objects across trials (“indirect trials”). We further investigated whether and how musical expertise modulates these two processes. We analyzed accuracy and reaction times (RTs) of direct and indirect trials in both groups. We reasoned that the musical and cross-modal memory demands of musicianship might modulate performance in the task and might thus reveal mechanisms that underlie the association and integration of visual information with musical information. Although musicians showed a higher overall memory accuracy, non-musicians’ accuracy was well above chance level in both trial types, thus indicating a significant ability to associate and integrate musical with visual information even in musically untrained subjects. However, non-musicians showed shorter RTs in indirect compared to direct trials, whereas the reverse pattern was found in musicians. Moreover, accuracy of direct and indirect trials correlated significantly in musicians but not in non-musicians. Consistent with previous accounts of visual associative memory, we interpret these findings as suggestive of at least two complimentary mechanisms that contribute to visual-melodic memory integration. (I) A default mechanism that mainly operates at encoding of complex visual-melodic associations and that works with surprising efficacy even in musically untrained subjects. (II) A retrieval-based mechanism that critically depends on an expert ability to maintain and discriminate visual-melodic associations across extended memory delays. Future studies may investigate how these mechanisms contribute to the everyday experience of music-evoked memories.

## Introduction

Music and memory are intimately related. Listening to specific melodies can evoke multiple memories, even in musical laypersons. Besides regulation of arousal and mood, expression of social relatedness and achievement of self-awareness, previous research identified the association of music with memories as one motivation for music listening (Laukka, 2007; Lonsdale and North, 2011; Schäfer et al., 2013). Accordingly, current theories of music processing postulate mechanisms by which music is associated with non-musical memories (Peretz and Coltheart, 2003; Koelsch, 2015; Jäncke, 2019). A modular model posits that melodic information may be stored in a musical lexicon module that may link with non-musical associative memories depending on contextual demands (Peretz and Coltheart, 2003). These memories may be episodic and may have autobiographical significance for the listener. Musical material may thereby reactivate contextual information from learning and may trigger corresponding emotional responses (Koelsch, 2015; Jäncke, 2019). Studies have moreover shown that music is particularly powerful in evoking non-musical perceptual details of previously experienced episodes (Janata et al., 2007; Belfi et al., 2016). These theories and results therefore suggest that music not only efficiently links with non-musical information, but may also integrate distinct non-musical information into complex cross-modal representations. However, how these representations are formed in the human brain has rarely been investigated. It appears possible that these representations are distinct from non-musical associative memories, as the cerebral organization of musical memory differs from other memory modalities (Groussard et al., 2010b,c; Finke et al., 2012; Esfahani-Bayerl et al., 2019).

Here, we investigated the ability of human subjects to associate visual objects with melodies and to integrate these associations into more complex representations where melodies are linked with two separately learned visual objects. We studied non-musicians as well as professional musicians. We reasoned that active musicianship might modulate the underlying memory processes, since active music making has been shown to critically depend on learning and memory (Wan and Schlaug, 2010; Brown et al., 2015; Altenmüller and Furuya, 2016). Musicians frequently learn entire musical pieces or a repertoire of tunes and know their musical structure, melodies and harmonic progressions by heart. Musical performance moreover puts particular demands on cross-modal memory abilities, as it requires an association of visual notation with sounds and corresponding motor responses (Jäncke, 2019). In line with this, music making, in particular at a professional level, has been shown to be associated with changes in brain areas that are involved in learning and memory. For instance, gray-matter volumes in the hippocampus differ between musicians and non-musicians and increase with the amount of musical expertise (Groussard et al., 2014). In addition, stronger hippocampal activation was found in musicians compared to non-musicians in a musical familiarity task which might further indicate specific memory abilities in musicians (Groussard et al., 2010a).

In our study, we used a variant of the associative inference paradigm, i.e., a task that has previously been used in behavioral and fMRI studies of visual associative memory (Preston et al., 2004; Zeithamova and Preston, 2010; Zeithamova et al., 2012a; Pajkert et al., 2017; Shing et al., 2019). This task assesses a subjects’ ability to associate and memorize pairs of items (e.g., item “A” paired with item “B”) that either overlap with pairs of items in other trials (e.g., item “B” also paired with item “C”) or not (e.g., item “D” paired with item “E”). Importantly, it also assesses a subjects’ ability to build integrated representations across related stimulus pairs (i.e., across A-B and B-C pairs), The underlying process is called memory integration (Zeithamova et al., 2012b; Schlichting and Preston, 2015). This cognitive faculty is a major prerequisite for building networks of interrelated memory items and for various non-mnemonic cognitive functions (Zeithamova et al., 2012b, 2019; Schlichting and Preston, 2015; Duncan and Schlichting, 2018). Previous studies have shown that analysis of accuracy and reaction times (RTs) of behavioral responses in associative inference tasks allows for inferences on the timing and nature of the corresponding integration process (Schlichting et al., 2014; Pajkert et al., 2017; Shing et al., 2019). In the visual-melodic variant used here, “A” and “C” stimuli were always visual objects in distinct trials that were linked by a common melodic “B” stimulus. Participants were thus required to memorize overlapping object-melody pairs and to form an integrated and more complex cross-modal representation where a melody links with visual objects across trials. We analyzed accuracy and RTs both for memory of object-melody associations *per se* (i.e., “direct trials”) and for memory integration across overlapping object-melody pairs (i.e., “indirect trials”). We expected significant performance differences between groups that might reveal basic mechanisms underlying the association and integration of visual with musical information.

## Materials and methods

### Participants

A total of 60 participants was included in the study, 30 professional musicians and 30 non-musicians (Table 1). The professional musicians either studied at a music university or music school or had completed their studies and worked as instrumental teachers, freelance and orchestra musicians. All musicians were instrumental musicians (string instruments *n* = 12; keyboard instruments *n* = 6; woodwind instruments *n* = 5; brass instruments *n* = 3; plucking instruments *n* = 4). The non-musician group consisted of 30 participants without or with minimal extracurricular musical activity. Six of these participants reported that they had played or tried a musical instrument or had sung in a school choir for 6 months to 2.5 years. However, musical activity was abandoned at least 10 years prior to study participation. Non-musicians were recruited from staff and students of the Charité – Universitätsmedizin Berlin and from other Berlin universities. One additional non-musician was excluded from data analysis, since her memory accuracy in direct trials was below chance level.

**Table 1:** Demographics and musical activity of the musician and non-musician groups.

	Musicians (*n* = 30)	Non-musicians (*n* = 30)	Test statistic and *p* value
Sex (female/male)	14/16	14/16	χ^2^(1) = 0, *p* = 1.00
Age (y)	25.40 ± 5.87	26.37 ± 4.78	*W* = 377.5, *p* = 0.286
Years of education (y)	15.37 ± 2.34	15.82 ± 1.66	*W* = 404, *p* = 0.498
Reasoning – LPS subtest number 3 (T Score)	61.83 ± 5.94	59.67 ± 6.81	*W* = 537.5, *p* = 0.185
MBEA Scale subtest (%)	94.89 ± 5.98	87.67 ± 7.12	*W* = 727.5, *p* < 0.001
Daily music listening^a^ (h)	1.53 ± 1.17	1.89 ± 1.64	*W* = 390.5, *p* = 0.501
Concert visits^b^	27.83 ± 28.61	6.80 ± 16.25	*W* = 785.5, *p* < 0.001
Age of first instrumental practice (y)	5.67 ± 1.99	-	
Total years of instrumental practice (y)	19.05 ± 5.54	0.22 ± 0.59	
Accumulated instrumental practice time (h)	17,448 ± 9,005	14 ± 44	
General average weekly practice time^c^ (h)	16.02 ± 7.62	0.22 ± 0.44	
Current average weekly practice time^d^ (h)	24.05 ± 12.11	–	–
Absolute pitch (yes/no)	9/21	–	–

Values are given as means with standard deviations or as frequencies. χ^2^, chi-square test; y, years; h¸ hours; W, Wilcoxon rank-sum test; LPS, Leistungsprüfsystem; MBEA, Montreal Battery of Evaluation of Amusia.

a Average hours of daily music listening during the last 12 months.

b Average number of attended concerts or music events during the last 12 months.

c Average practice time per week across all decades of musical activity.

d Average practice time per week during the last 12 months.

No participant reported a history of neurological or psychiatric diseases, hearing deficits or significant visual impairments. The musician and non-musician groups were matched for sex, age and educational level (Table 1). Both groups were comparable in terms of non-verbal intelligence as measured with a logical reasoning task (Subtest 3 of the test battery Leistungsprüfsystem LPS; Horn, 1983). The Scale Subtest of the Montreal Battery of Evaluation of Amusia (MBEA; Peretz et al., 2003) was used to screen for amusia and assess basic music perceptual abilities. Although musicians outperformed non-musicians in this test, all non-musician participants scored within the normal range. All participants gave written informed consent before participation in the study and were paid for participation. The study was approved by the local Ethics Committee of the Charité – Universitätsmedizin Berlin and was conducted in conformity with the Declaration of Helsinki. Determination of sample size was based on previous studies using associative inference paradigms (Pajkert et al., 2017; Schlichting et al., 2017; Shing et al., 2019) and on studies of musical memory of musicians and non-musicians (Groussard et al., 2010a; Gagnepain et al., 2017). A *post hoc* sensitivity analysis was conducted using G*Power 3 (Faul et al., 2007) for ANOVA analyses of accuracy and RTs (between, within and between-within interactions, respectively), which indicated that medium to large effect sizes (Cohen’s *f* = 0.37/η^2^ = 0.12) could be detected with the given *N* = 60 participants, an α = 0.05 and a power of 0.80.

### Assessment of musical activity

Indices of musical activity were assessed using a short questionnaire (MusA; Fernholz et al., 2019). This questionnaire covers both music reception (i.e., music listening, concert attendance) and active musical practice (i.e., instrument group, years of musical activity, weekly practice time). The weekly average time of musical practice was assessed for each age decade (i.e., 0–10 years, 11–20 years, 21–30 years etc.). Additionally, weekly average time of music making during the last 12 months and total years of instrumental practice were measured. The variables assessed *via* the MusA were used to calculate further indices of musical activity. The general average practice time across all decades was determined by calculating the mean of the weekly average time of playing music for each age decade. The cumulative practice time on the instrument was calculated by combining total years of instrument playing with weekly practice times. In addition, we assessed musical activity variables that were not covered by the questionnaire by using a short personal interview (age of first instrumental practice, played instruments, absolute pitch). Descriptive information of the indices of musical activity is reported in Table 1.

### Visual-melodic associative inference task

#### Stimuli

In our task, both visual and musical stimuli were used. Visual stimuli were taken from the Bank of Standardized Stimuli (BOSS Phase II; Brodeur et al., 2014) and consisted of 331 colored images of everyday objects (e.g., tools, food, clothes, toys etc.). Musical stimuli involved 43 melodies played in a piano voice, without orchestration and lyrics, even if the original piece included lyrics. Melodies were taken from various genres, such as classical music, jazz, folk songs (from non-German speaking countries) or themes from older TV series or movies (see Supplementary Table 1). We aimed to include melodies that are unlikely to be associated with visual information (e.g., themes from popular movies) or with autobiographical memories (e.g., children’s songs, pop songs, major themes from classical music). Only melodies were included that were not on web-based lists of canonical works of classical music. Melodies had a mean duration of 7 s (Range: 5–10 s). Melodies had a different duration in order to preserve the musicality of the stimuli and avoid cutting the melody in the middle of a phrase or playing them at a much faster or slower tempo. We further verified that length of melodies did not predict accuracy in direct trials (see Supplementary Table 2).

Musical stimuli were evaluated for familiarity in a pilot experiment with *n* = 19 participants with different levels of musical training [*n* = 3 non-musicians, *n* = 5 inactive amateur musicians (age of first instrumental practice: *M* = 8.00, *SD* = 1.73, range: 6–9 years; total years of instrumental practice: *M =* 7.75, *SD* = 2.06, range: 5–10 years), *n* = 4 active amateur musicians (age of first instrumental practice: *M* = 5.25, *SD* = 1.00, range: 6–8 years; total years of instrumental practice: *M =* 17.00, *SD* = 5.00, range: 12–22 years) and *n* = 7 professional musicians (age of first instrumental practice: *M* = 5.14, *SD* = 1.21, range: 4–7 years; total years of instrumental practice: *M* = 17.67, *SD* = 2.80, range: 15–23 years)]. Three additional melodies were pre-rated, but excluded from the experiment since they had a high level of recognition (i.e., between 21 and 26% of participants recognized them). During the experiment, participants were asked to verbally report if they knew the melody. Seven musicians (23.33%) reported to recognize one or two melodies, one non-musician (3%) reported to recognize one melody.

#### Procedure

The experiment was performed using Presentation® software (Version 18.1, Neurobehavioral Systems, Inc. Berkeley, CA, United States) and was conducted in a quiet room. The duration of the experiment was approximately 50 min. Musical stimuli were presented *via* external speakers and participants could adapt the volume to their needs. Prior to the experiment, participants were instructed about the task with example stimuli and received a short training with a small number of trials. Melodies and objects of the training session were not included in the experiment. The experimenter repeated instructions if necessary and ensured full comprehension of instructions before the experiment was started.

**Figure 1:**
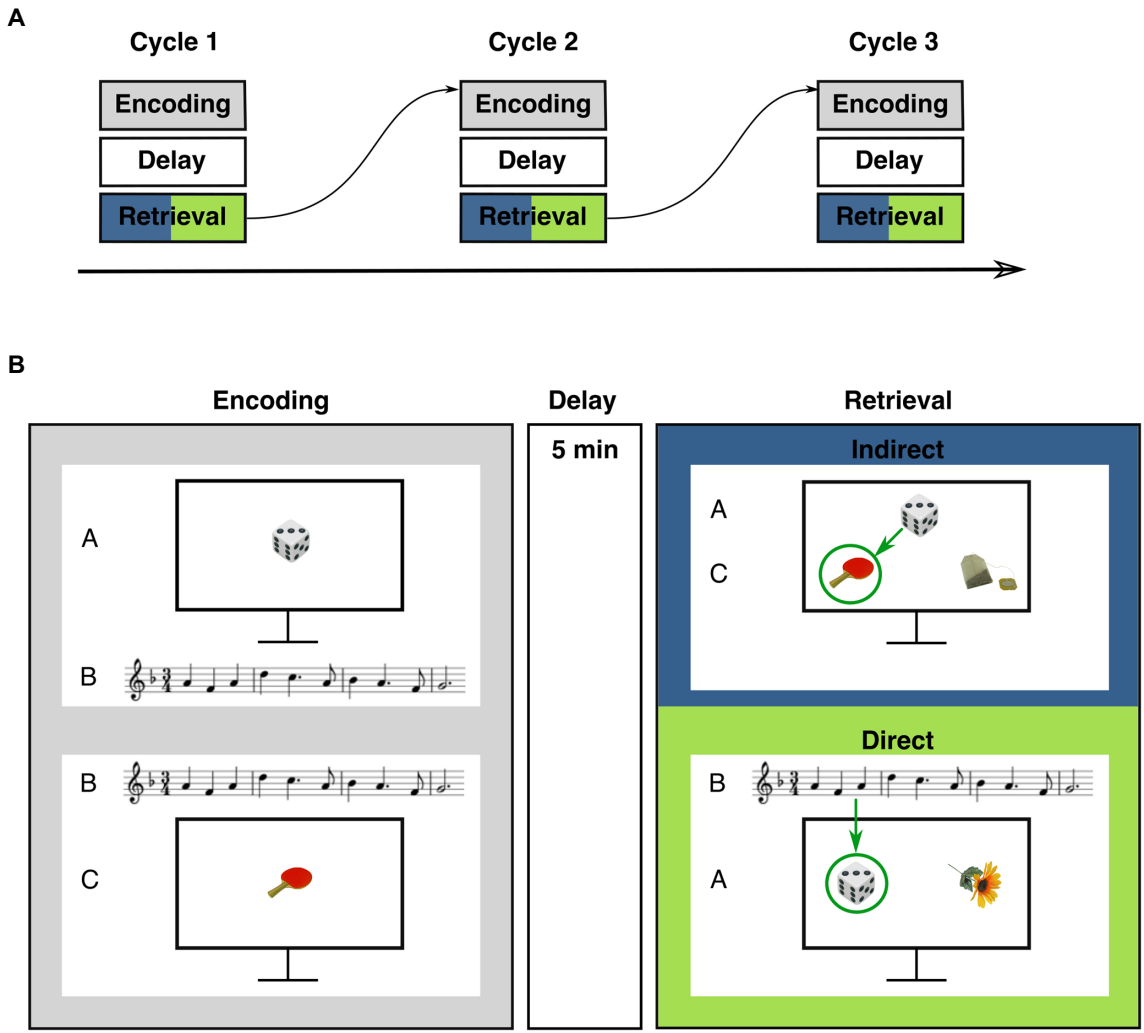
Procedure and example stimuli of the musical associative inference task. **(A)** Task structure: The experiment consisted of three alternating encoding and retrieval blocks that were separated by a delay of 5 min. **(B)** Example stimuli of encoding and retrieval blocks. During the encoding block, participants studied overlapping pairs of objects and melodies (AB-/BC-pairs) and non-overlapping DE-pairs (not shown). Note the overlapping melody of AB-/BC-trials. At retrieval, participants were tested on studied direct trials (AB-, BC-, and DE-pairs) and on indirect trials (inferential AC-pairs). Green arrows indicate the correct choice.

The task consisted of alternating encoding and retrieval blocks. Encoding blocks were followed by an unfilled memory delay of 5 min (Figure 1A). Then, the corresponding retrieval block started. The experiment consisted of three cycles with one encoding and one retrieval block in each cycle. During the encoding blocks, participants studied pairs of objects and melodies. Some of the pairs shared a melody, i.e., objects from distinct encoding trials were paired with the same melody and were thus indirectly associated through this melody. Some of the object-melody pairs did not share a melody with another trial. During the subsequent retrieval blocks, participants were tested both for memory of the object-melody pairs (“direct trials”) and for inferential associations, i.e., associations between objects that were indirectly linked *via* a common melody (“indirect trials”). Objects and melodies were unique to each cycle.

Each encoding block consisted of 18 trials with object-melody pairs. Like in previous studies with associative inference paradigms (e.g., Preston et al., 2004; Zeithamova and Preston, 2010; Pajkert et al., 2017; Shing et al., 2019) these pairs were termed AB-, BC-and DE-pairs. During each encoding block, six AB-, six BC-and six DE-trials were presented. In AB-and BC-trials, A-and C-stimuli were always objects and the B-stimulus always a melody. AB-and BC-trials were overlapping, i.e., they shared the same melody (B-stimulus) so that two distinct objects (A and C) were associated with a common melody. Participants were presented an object (A) on the computer screen and a melody (B) was played at the same time. After two to four trials, the same melody (B) was played again, but was now paired with another object (C). In addition, DE-trials were presented, consisting of one object (D-stimulus) and one melody (E-stimulus). These stimuli were non-overlapping, i.e., they did not share a melody with other trials. The combination of objects and melodies was trial-unique and pseudo-randomized for each participant. Within each encoding block, the order of the trials was pseudo-randomized using the program Mix (Van Casteren and Davis, 2006). AB-pairs were always presented before their corresponding BC-pairs, with two to four trials in between. These intervening trials were either AB-trials or BC-trials from other overlapping AB-/BC-pairs or DE-trials. DE-trials were intermixed with AB-and BC-trials and included in the design to establish a minimum distance between AB-and BC-trials and to increase uncertainty about occurrence and timing of BC-trials. Presentation time of each pair was determined by the length of the respective melody. In order to ensure that participants focused on the presented stimuli, participants were asked how they liked the melodies after each trial. Responses were given on a five-point Likert scale (1 = not at all, 5 = very much). Trials were terminated after a response was given. The inter-trial interval was 5 s.

Each retrieval block consisted of 24 trials (6 AC-, 6 AB-, 6 BC-, and 6 DE-trials). To clarify the fundamental difference between retrieval trial types, AC-trials were termed ‘indirect trials’ and AB-, BC-, and DE-trials were collectively termed ‘direct trials’ (Schlichting et al., 2014; Pajkert et al., 2017; Shing et al., 2019). In each indirect (AC-) trial, one A-stimulus (i.e., an object) was presented at the top of the screen (Figure 1B). Two C-stimuli were shown at the bottom of the screen, one representing the target object and one a foil object. Participants had to decide which of the two C-stimuli at the bottom had previously been presented with the same melody as the A-stimulus. Thus, participants had to infer which of the objects at the bottom of the screen shared an indirect relation with the object at the top *via* a common B-stimulus (i.e., a melody). Participants indicated their choice *via* button press. Subsequently, memory for direct associations was tested, i.e., memory for object-melody associations as presented during encoding. All 18 AB-, BC-, and DE-stimuli of the respective cycle were tested. In these direct retrieval trials, two objects (i.e., either two A-, C-or D-stimuli) were shown in the middle of the computer screen. At the same time, a melody was played (either a B-or E-stimulus). Participants had to indicate by button press which of the two objects had previously been paired with the melody (Figure 1B).

Indirect (AC-) trials were always presented at the beginning of a retrieval block. Then, direct trials were presented (i.e., AB-, BC-, and DE-trials). This design was chosen to avoid relearning of AB-and BC-trials before testing of AC-pairs. The order both of indirect and direct trials was randomized. Presentation of the stimuli was terminated by the key press of the participants. To avoid differences in familiarity of target and foil stimuli, all foils were taken from other pairs of the same cycle. In each indirect and direct retrieval block, stimuli were always from the preceding encoding block of the same cycle.

### Data analysis

#### Main analyses

For the musical associative inference task, we analyzed accuracy, i.e., the percentage of correct responses for each trial type, in each participant. We further analyzed reaction times (RTs) of correctly answered trials for each trial type. Medians were used to describe individual average RTs for each trial type. Due to the limited number of trials per cycle and trial type, data were averaged across cycles. Since most of the variables of interest were not normally distributed, a non-parametrical statistical approach was used throughout. Analyses were performed using R Studio (version 3.6.3; R Core Team, 2020).

First, accuracy in indirect and direct trials was compared against chance level (i.e., 50% correct answers) in both groups using a Wilcoxon signed rank test. Rank-biserial correlations (*r*) were calculated as measures of effect size. Then, effects of group (between-factor) and trial type (within factor) on accuracy and RTs were analyzed with a repeated measures design for non-normal data using the package MANOVA.RM (Friedrich et al., 2019, 2021). With this package, robust test statistics can be calculated, even when the basic assumptions for parametric approaches (i.e., normal distribution, equal covariances) are violated. We calculated Wald-type statistics (WTS) with permuted *p*-values to account for non-normal data distribution. Significant interactions were followed by pairwise comparisons. For post-hoc comparison of within factors (i.e., trial type), one-way repeated measure ANOVAs were performed with the RM function of the MANOVA.RM package. For post-hoc analysis of group differences, we used the package GFD (Friedrich et al., 2017) to calculate WTS combined with a permutation procedure for *p*-values. The Bonferroni-Holm correction (Holm, 1979) was used to adjust for multiple comparisons in the post-hoc analysis. As measures of effect size, partial eta squared (η^2^) was calculated. Note that we calculated parametric effect sizes, since non-parametric measures of effect size for ANOVA-type analyses are currently not available. For post-hoc tests, we additionally calculated rank-biserial correlations (*r*) for non-parametric effect sizes. All effect sizes were calculated using the package effectsize (Ben-Shachar et al., 2020).

Based on previous studies using a visual associative inference paradigm (Pajkert et al., 2017; Shing et al., 2019), we analyzed correlations between accuracy in indirect and direct trial types by using Kendall’s τ. For comparison of demographic data and musical activity variables across groups, Wilcoxon rank-sum tests were calculated. The significance level was set at *p* < 0.05.

#### Exploratory analyses

In addition to main analyses, we performed a detailed analysis of performance of related direct and indirect trials (i.e., AC-trials and their corresponding AB-and BC-trials; see 3.4.1 for detailed description) in both groups by using the rm-function of the package MANOVA.RM (Friedrich et al., 2019, 2021). As for the main ANOVA analyses, WTS with permuted *p*-values were calculated to account for non-normal data distribution. Partial eta squared (η^2^) and rank-biserial correlations (*r*) were reported for effect sizes.

## Results

### Main analyses

#### Accuracy

We first analyzed differences in accuracy between direct trials with an overlapping melody (i.e., AB-and BC-trials) and non-overlapping direct trials (i.e., DE-trials) and conducted a repeated measures ANOVA for non-normal data with group (musicians, non-musicians) as between-factor and trial type as within-factor (AB-/BC-trials, DE-trials). Since the main effect of direct trial types [WTS(1) < 1, *p* = 0.393, η*2* = 0.01] and the interaction between group and trial type [WTS(1) = 3.23, *p* = 0.076, η*2* = 0.05] was not significant, all direct trials (i.e., AB-, BC-and DE-trials) were pooled for further analysis, like in previous studies (Zeithamova and Preston, 2010; Pajkert et al., 2017).

**Figure 2:**
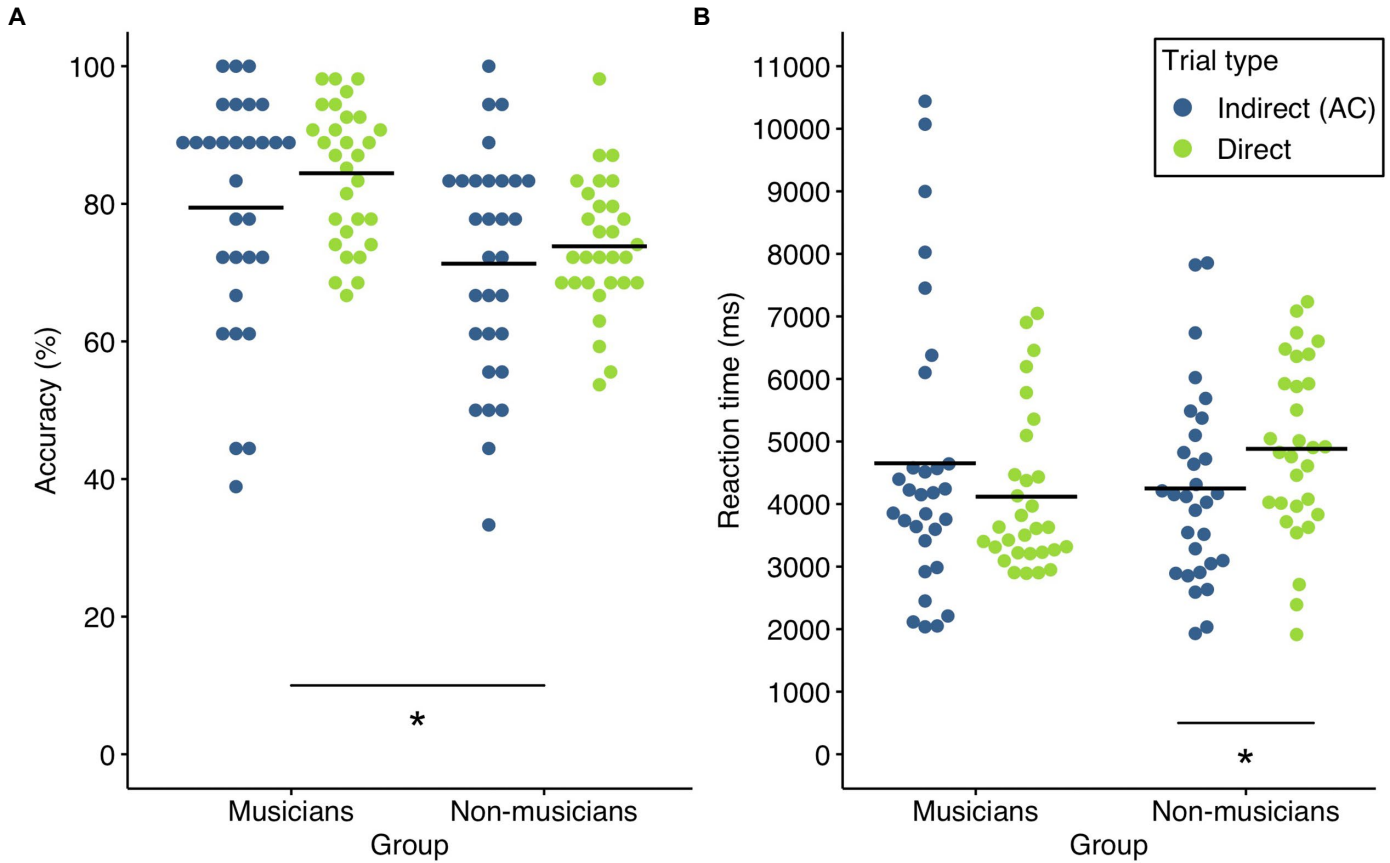
Accuracy and reaction times of both groups. **(A)** Accuracy in indirect (blue) and direct (green) trial types in musicians and non-musicians. There was a significant group effect (*p < 0.05) **(B)** Reaction times of correctly answered indirect (blue) and direct (green) trial types in musicians and non-musicians. There was a significant interaction of group × trial type. The asterisk denotes the significant pairwise comparisons (*p < 0.05 after Bonferroni-Holm correction). Solid lines represent the respective mean.

Accuracy of indirect and direct trials in both groups is shown in Figure 2. In a first step, we checked whether both groups performed above chance level (i.e., 50% correct answers) in indirect and direct trials using a Wilcoxon signed rank test. For both trial types, accuracy was significantly above chance level in musicians (indirect trials: *M* = 79.44%, *SD* = 17.08%, *W* = 457.5, *p* < 0.001, *r* = 0.97; direct trials: *M* = 84.44%, *SD* = 9.79%, *W* = 465, *p* < 0.001, *r* = 1.00) and non-musicians (indirect trials: *M* = 71.29%, *SD* = 16.38%, *W* = 367, *p* < 0.001, *r* = 0.94; direct trials: *M* = 73.83%, *SD* = 9.61%, *W* = 465, *p* < 0.001, *r* = 1.00). On an individual level, all of the included musician and non-musician participants had a performance higher than 50% in direct trials.

Accuracy was then analyzed using a repeated measures ANOVA for non-normal data with group (musicians, non-musicians) as between-factor and trial type (indirect, direct) as within-factor. There was a significant group difference [WTS(1) = 10.49, *p* = 0.002, η^2^ = 0.15]. Averaged across trial types, musicians (*M* = 81.94%, *SD* = 12.12%) performed superior to non-musicians (*M* = 72.56%, *SD* = 10.25%). There was however no significant effect of trial type [WTS(1) = 3.47, *p* = 0.072, η^2^ = 0.06] or interaction of trial type and group [WTS(1) < 1, *p* = 0.545, η^2^ = 0.006]. Although musicians outperformed non-musicians in both trial types, non-musicians were apparently able to efficiently associate and memorize object-melody pairs (direct trials) and to integrate these pairs into more complex representations (indirect trials).

#### Reaction times

RTs of indirect and direct trials in both groups are shown in Figure 2. For analysis of RTs of correctly answered trials, a repeated measures ANOVA with group (musicians, non-musicians) as between-factor and trial type (indirect, direct) as within-factor was calculated. There was no significant main effect of group [WTS(1) < 1, *p* = 0.623, η^2^ = 0.004] or trial type [WTS(1) < 1, *p* = 0.828, η^2^ < 0.001], indicating that non-musicians were generally as fast as musicians in retrieving associations between objects and melodies and that RTs were not generally shorter in one of the trial types. The interaction effect of group and trial type however was significant [WTS(1) = 7.1, *p* = 0.009, η^2^ = 0.11]. We thus compared the respective levels of the factors trial type and group (corrected for four pairwise comparisons). Post-hoc tests showed trial type differences for non-musicians [WTS(1) = 9.34, *p* = 0.016, η^2^ = 0.24, *r* = 0.54]. RTs were significantly shorter in indirect trials (*M* = 4,249 ms, *SD* = 1,526 ms) compared to direct trials (*M* = 4,882 ms, *SD* = 1,392 ms). In the musician group, the post-hoc test did not reveal any difference between RTs in indirect (*M* = 4,652 ms, *SD* = 2,276 ms) and direct trials [*M* = 4,118 ms, *SD* = 1,249 ms; WTS(1) = 1.917, *p* = 0.362, η^2^ = 0.06, *r* = 0.20]. Post-hoc tests between the two groups did not show significant differences for indirect [WTS(1) < 1, *p* = 0.427, η^2^ = 0.01, *r* = 0.05] or direct trials [WTS(1) = 5.02, *p* = 0.081, η^2^ = 0.08, *r* = 0.380] after correction for multiple comparisons.

Consistent with previous studies of memory integration in healthy humans and patients with hippocampal damage (Schlichting et al., 2014; Pajkert et al., 2017; Shing et al., 2019), we reasoned that the different RT patterns might reflect different strategies for memory integration in the two groups. Shorter RTs in indirect trials compared to direct trials in the non-musician group might suggest that non-musicians build integrated and complex associations already during the encoding phase of the task. These representations may be formed as soon as an object-melody (i.e., BC) pair is encoded that shares a melody with a preceding object-melody (i.e., AB) pair. The resulting object-melody-object (ABC-) triplet may then be represented across the memory delay until the retrieval phase of the task. In this framework, the RT pattern in the musician group would suggest a distinct and more retrieval-based strategy with musicians memorizing object-melody pairs separately until the retrieval phase of task.

#### Correlation of accuracy between direct and indirect trials

Following the rationale of previous studies (Pajkert et al., 2017; Shing et al., 2019), we further investigated our hypothesis of distinct behavioral strategies and analyzed the correlational pattern between accuracy in indirect trials (AC) and overlapping direct trial types (AB and BC) in both groups. If musicians indeed based their AC-decisions at retrieval mainly on knowledge of separately memorized AB-and BC-pairs, a correlation between accuracy in AC-trials with accuracy of AB-and BC-trials should be expected. In non-musicians, however, no or weaker correlations should be expected, since integrated ABC-triplets may already be formed during encoding. AC-decisions at retrieval would then be less dependent on separate memory of the corresponding AB-and BC-pairs.

**Figure 3:**
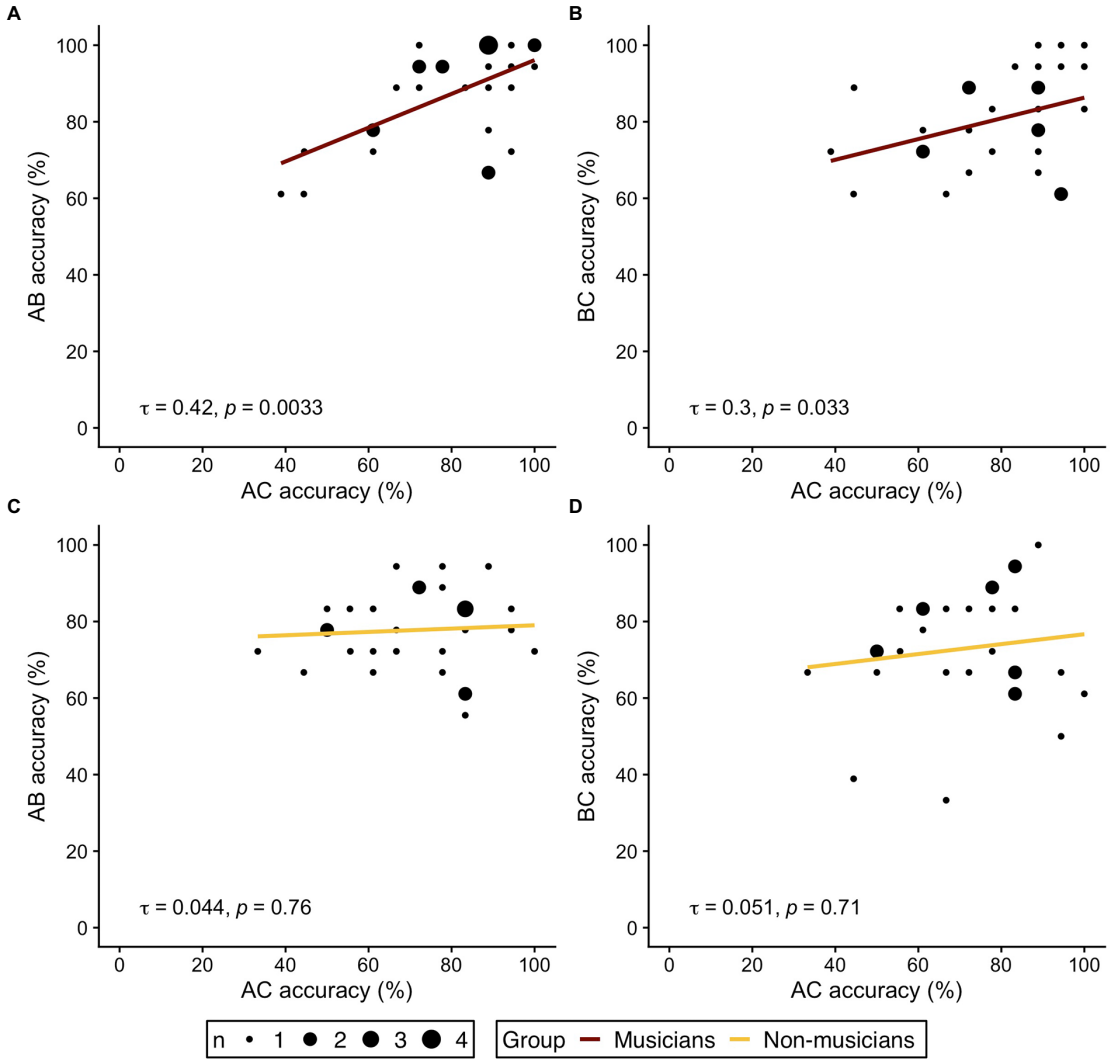
Correlations of indirect trials (AC accuracy on the respective x axis) and direct trials (AB, BC accuracy on the respective y axis) in musicians (**A,B**, red) and non-musicians (**C,D**, yellow). **(A,C)** Correlation of AC- and AB-trials. **(B,D)** Correlation of AC- and BC-trials. τ refers to the correlation coefficient from Kendall’s τ. Dot size represent the number of identical values.

Figure 3 displays the correlation plots for both groups and the respective bivariate correlations. In the musician group, correlation analyses revealed significant correlations between AC accuracy and performance in the underlying direct trial types (AC-AB: τ = 0.42, *p* = 0.0033; AC-BC: τ = 0.3, *p* = 0.033). No correlation between AC performance and accuracy in AB-or BC-trials was observed in non-musicians (AC-AB: τ = 0.044, *p* = 0.76; AC-BC: τ = 0.051, *p* = 0.71). The results of the correlation analysis therefore corroborate the hypothesis of different behavioral strategies for memory integration in musicians and non-musicians.

### Exploratory analyses

**Figure 4:**
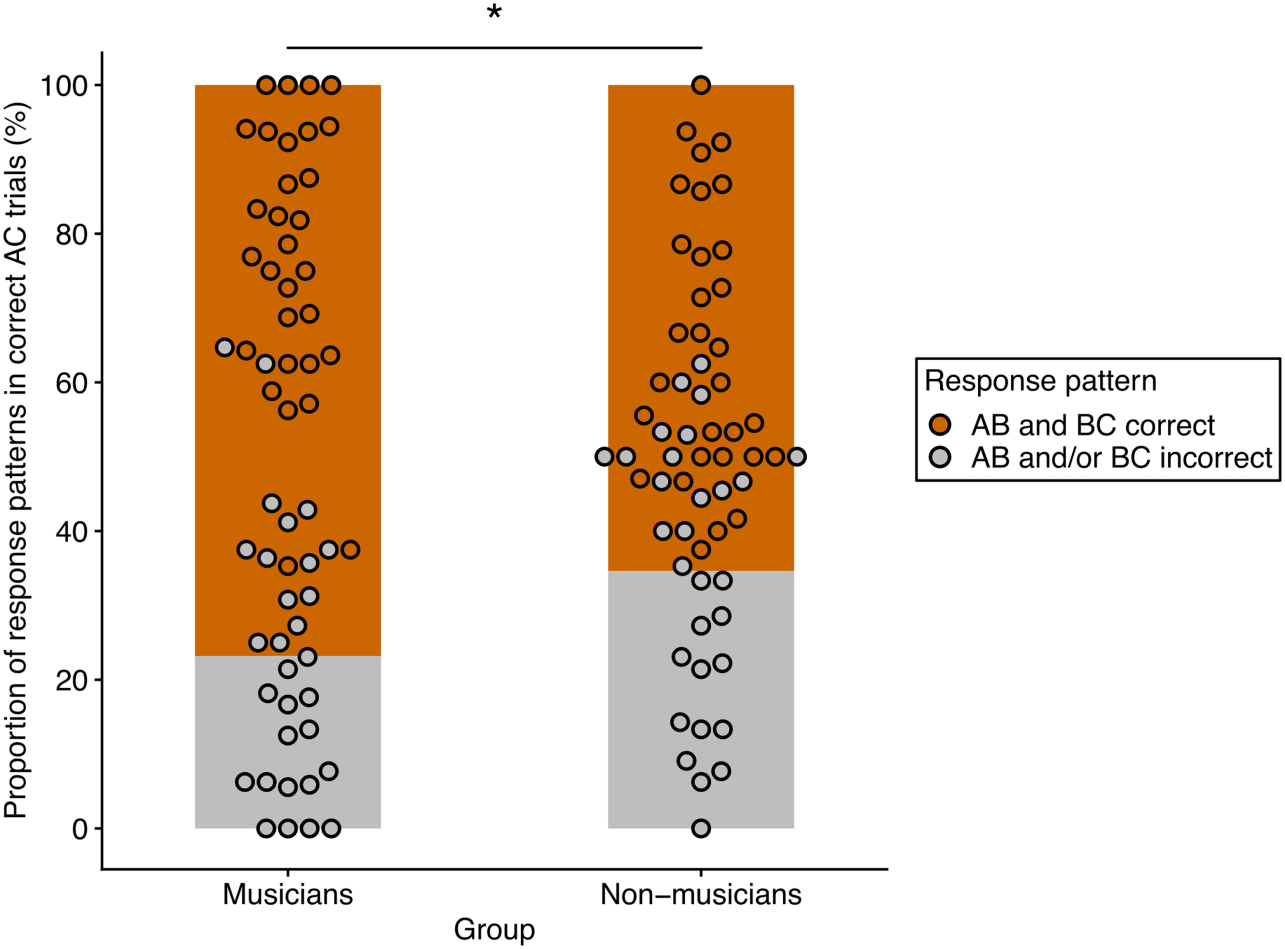
Relative frequencies of the two response patterns in musicians and non-musicians. AB and BC correct (orange) refers to the relative percentage of correctly answered AC-trials in which the corresponding AB-and BC-trials of the same overlapping ABC-triplet were also correctly answered. AB and/or BC incorrect (gray) refers to the relative percentage of correctly answered AC-trials in which the corresponding AB-or BC-or both trials of the same overlapping ABC-triplet were incorrectly answered. There was a significant effect of response patterns and a significant interaction of response pattern and group. The asterisk denotes the significant pairwise comparison of response patterns between groups (*p < 0.05 after Bonferroni-Holm correction).

For a final test of the hypothesis of different strategies underlying memory integration between groups, we analyzed accuracy of the corresponding indirect and direct trials. In a first step, we took AC-trials that were correctly answered at retrieval and checked whether the corresponding AB-and BC-trials were also correct. This resulted in two response patterns: (1) Correct AC-trials, for which the corresponding AB-and BC-trials were also correct. (2) Correct AC-trials for which the corresponding AB-or BC- trials or both were incorrect. Relative percentages of these two response patterns were then calculated for each participant by dividing the number of each response pattern by the number of correctly answered AC-trials. Figure 4 displays the relative proportion of response patterns in both groups.

We then calculated a repeated measures ANOVA for non-normal data with group as between-factor and response pattern as within-factor. There was a significant main effect of response pattern [WTS(1) = 83.15, *p* < 0.001, η^2^ = 0.59] and a significant interaction effect of group and response pattern [WTS(1) = 6.11, *p* = 0.016, η^2^ = 0.10]. The main effect of group was not significant [WTS(1) = 1.65, *p* = 0.21, η^2^ = 0.03]. Post-hoc analysis (corrected for four pairwise comparisons) revealed response pattern differences both for musicians [WTS(1) = 68.12, *p* = 0.004, η^2^ = 0.70, *r* = 0.94; AB and BC correct: *M* = 76.81%, *SD* = 17.79%; AB and/or BC incorrect: *M* = 23.19%, *SD* = 17.79%] and non-musicians [WTS(1) = 21.79, *p* = 0.004, η^2^ = 0.43, *r* = 0.73; AB and BC correct: *M* = 65.37%, *SD* = 18.04%; AB and/or BC incorrect: *M* = 34.63%, *SD* = 18.04%]. Not surprisingly, the underlying AB-and BC-trials were correct in the majority of correctly answered AC-trials in both musicians and non-musicians. However, when we compared response patterns between groups, we found significant differences for both response patterns [AB and BC correct: WTS(1) = 6.11, *p* = 0.035, η^2^ = 0.10, *r* = 0.37; musicians: *M* = 76.81%, *SD* = 17.79%; non-musicians: *M* = 65.37%, *SD* = 18.04%; AB and/or BC incorrect: WTS(1) = 6.11, *p* = 0.035, η^2^ = 0.10, *r* = 0.37; musicians: *M* = 23.19%, *SD* = 17.79%; non-musicians: *M* = 34.63%, *SD* = 18.04%]. Thus, in correct AC-trials, musicians had a higher percentage of trials in which both the corresponding AB-and BC-pairs were also correct than non-musicians. Non-musicians had a higher percentage of correct AC-trials in which the corresponding AB-or BC-trials or both were incorrect. Apparently, non-musicians could still make correct AC-decisions, even in trials where they did not correctly remember the underlying AB-and BC-pairs.

## Discussion

We investigated how musicians and non-musicians build associations between visual objects and melodies and integrate these associations into more complex memory representations. Using an associative inference task with visual and musical stimuli, we compared accuracy and RTs of professional musicians and non-musicians for memory of simple visual-melodic associations (direct trials) and for more complex associations in which melodies link otherwise unrelated visual object information (indirect trials). Accuracy of both musicians and non-musicians was above chance level in both trial types, indicating that participants could reliably memorize and retrieve associations of objects with melodies and were able to link distinct and previously unrelated visual information into integrated memory representations *via* association with a common melody. Although musicians outperformed non-musicians in direct and indirect trials, our results show that the process of building complex and indirect links between music and non-musical memories can happen with surprising efficacy even in musically untrained subjects. Our findings however suggest that musicians and non-musicians use different strategies for integration of visual with musical information.

Consistent with the superior overall performance of musicians in our study, musicians have been found to have superior auditory memory compared to non-musicians, not only for musical but also for non-musical auditory stimuli (Cohen et al., 2011). In both musicians and non-musicians, however, auditory memory was inferior to visual memory, which was comparable between groups (Cohen et al., 2011). Similarly, a meta-analysis found that, compared to non-musicians, musicians have a better performance in memory tasks, with a small effect for long-term memory and medium effect sizes for short-term and working memory tasks (Talamini et al., 2017). Better memory performance was however dependent on stimulus type. For short-term and working memory tasks, the memory advantage of musicians was large for tonal stimuli, moderate for verbal stimuli and small or null when visuospatial stimuli were involved. In a more recent study, visual and auditory short-term memory in musicians and non-musicians was compared using different categories of stimuli (i.e., verbal, non-verbal with contour, non-verbal without contour; Talamini et al., 2021). Stimulus sequences with contour included up and down variations based on loudness (auditory condition) or luminance (visual condition). Musicians selectively performed better in both visual and auditory contour and auditory non-contour conditions, whereas memory performance in verbal conditions was comparable. These results suggest that musical activity preferentially trains memory domains that are closely related to musical skills. In line with this, research on other fields of expertise such as chess, medicine or mental calculations suggested that experts mainly have a domain-specific memory advantage for meaningful information within their field of expertise (Ericsson and Kintsch, 1995; Ericsson, 2017). It seems therefore likely that absolute performance differences across our two groups were at least partly driven by superior auditory memory in musicians rather than by a higher overall level of memory performance.

Several influential models of musical processing postulate mechanisms that associate musical with non-musical memories (Peretz and Coltheart, 2003; Koelsch, 2015; Jäncke, 2019). One important aspect of music-evoked memories is their perceptual richness. Previous studies have shown that music-evoked memories contain more perceptual details than memories evoked by visual stimuli such as faces (Janata et al., 2007; Belfi et al., 2016). Musical information may thus be particularly powerful in binding together distinct perceptual details in integrated and complex cross-modal memory representations. One experimental approach to address this issue is the associative inference paradigm. This memory task assesses a subjects’ ability to memorize pairs of items (e.g., item “A” paired with item “B”) that overlap with pairs of items in other trials (e.g., item “B” also paired with item “C”) presented during the encoding phase of the task. At retrieval, it assesses a subjects’ ability to build integrated representations across related stimulus pairs (i.e., across AB-and BC-pairs). To correctly perform in these ‘AC-trials’, overlapping AB-and BC-stimuli have to be linked at some point between encoding and retrieval *via* a B-stimulus, e.g., the melody in our experiment. Two complimentary processes have been postulated that may support memory integration (Zeithamova and Preston, 2010; Zeithamova et al., 2012b; Shohamy and Daw, 2015; Pajkert et al., 2017; Duncan and Schlichting, 2018). First, memory integration may be achieved by an integrative encoding mechanism (Shohamy and Wagner, 2008; Zeithamova and Preston, 2010; Zeithamova et al., 2012b; Duncan and Schlichting, 2018). This account posits that during encoding of BC-pairs, previously studied AB-pairs become reactivated *via* the overlapping B-stimulus. Thus, integrated ABC-representations are already formed during encoding and are readily available for later AC-decisions, since the underlying AB-and BC-pairs do not have to be retrieved separately (Zeithamova and Preston, 2010). A previous study showed that response times for untrained inferential associations could be as fast as for trained direct associations, lending support to the idea that integrated memories can already be constructed during the encoding phase of associative inference tasks (Shohamy and Wagner, 2008). Second, integration of distinct but related memories can also occur during retrieval. In this case, individual AB-and BC-pairs are memorized separately and are finally recombined for AC-decisions. This process has been termed recombination at retrieval (Zeithamova et al., 2012b) and appears to be more flexible, but may result in slower responses, since additional cognitive processes are necessary by the time of retrieval that are not required for retrieval of simple AB-and BC-associations (Shohamy and Daw, 2015). Neuroimaging studies suggest that the hippocampus supports memory integration both during encoding and retrieval (Zeithamova and Preston, 2010; Schlichting et al., 2014; Tompary and Davachi, 2017; Duncan and Schlichting, 2018; van Kesteren et al., 2020; Molitor et al., 2021). In line with these neuroimaging results, patients with lesions of the hippocampus and surrounding medial temporal lobe were found to have deficits in memory integration and in making inferences between items of overlapping memory networks (Pajkert et al., 2017; Nicolás et al., 2021).

Our data suggest that musicians and non-musicians used both integrative encoding and recombination at retrieval to build complex associations between musical and visual information–albeit with distinct preferences between groups. Non-musicians showed faster responses in correct indirect (AC-) trials compared to correct direct trials. We therefore assume that non-musicians mainly used an integrative encoding strategy in which they build a melodic link between A-and C-stimuli, i.e., an ABC-triplet that is formed when the BC-pair is presented. An integrated object-melody-object representation is therefore already formed during encoding and memorized for AC-decisions at retrieval. Early integration of AB-and BC-pairs into an ABC-representation during encoding likely makes non-musicians less dependent on precise knowledge of the underlying AB-and BC-pairs. Facing the limited expertise in maintaining precise musical information across extended memory delays, this strategy may prove beneficial in non-musicians and reduce the effort in coping with the demands of the task while preserving a complex cross-modal memory representation for future decisions. We therefore suggest that integrative encoding may represent a default mechanism for integration of visual with melodic information in musical laypersons.

Other than non-musicians, professional musicians seem to base their AC-decisions more on memory of the underlying AB-and BC-pairs, which they recombine flexibly at retrieval for AC-decisions. This may reflect that musical information has a higher relevance and is more closely related to personal behavior in professional musicians, who are often required to memorize melodies actively and consciously. For musicians, music must not only be recognized, but must also be reliably recalled and imitated. This is an important prerequisite for musical improvisation as well as for performances without sheet music. It has previously been proposed that memorizing melodies mostly involves chunking and consolidation of small musical ordered segments. Musical training may moreover foster acquisition of controlled and active learning strategies (e.g., chunking; Talamini et al., 2017). In our study, such an active learning strategy might have contributed to task performance, so that musicians could memorize and recombine the underlying chunks (i.e., pairs of melodies and objects; AB-and BC-pairs) more precisely and with less effort than non-musicians. We therefore assume that musicians not only rely on a default integrative encoding mechanism for visuo-melodic memory integration, but additionally have access to recombination at retrieval as a complimentary strategy, presumably allowing them to build associations between musical and non-musical information more deliberately and flexibly according to actual contextual demands.

Our study has important limitations. One limitation is the choice of musical stimuli. Although explicit recognition of melodies was rare in the musician group, a sense of familiarity for at least some of the melodies cannot be ruled out with certainty. This would be no surprise given that musicians have probably been exposed to a higher amount of musical material than musical laypersons. In line with this, musicians have been found to access familiar melodies more efficiently than non-musicians (Gagnepain et al., 2017). In addition, musicians are probably able to link familiar melodies to more detailed contextual and autobiographic information than non-musicians (Groussard et al., 2010a). Therefore, additional factors may have helped musicians in our study to correctly memorize and retrieve object-melody pairs. However, these factors do not argue against the use of recombination at retrieval as a predominant strategy for memory integration. A further limitation is the choice of the visual stimuli. These were simple and autobiographically irrelevant everyday objects and thus quite distinct from the complex multisensory input that usually makes up autobiographical memories. The significance of our findings for the obvious relationship of music with episodic and autobiographical memories remains therefore to be clarified.

Taken together, the findings reported here suggest that both musicians and non-musicians can associate melodies efficiently with visual information. However, musically trained and untrained individuals seem to differ in how they build integrated and more complex visuo-melodic representations. Our results suggest that integrative encoding is a default mechanism for integration of musical and non-musical stimuli that is available to a surprising degree even to musically untrained subjects. We speculate that this more passive and recognition-based mechanism may reflect a basic ability to intuitively attach sounds to objects with no or little conscious effort. We cannot be sure whether this is specific to music, but it appears possible that integrative encoding may contribute to the everyday experience of music-evoked memories. By contrast, recombination at retrieval seems to be a more active and recall-based strategy for memory integration that apparently depends on an expert ability to maintain and discriminate musical stimuli across memory delays. Future studies should investigate if distinct behavioral strategies in musicians and non-musicians depend on distinct neural substrates. Moreover, it will be important to investigate whether visual-melodic memory integration persists across extended memory delays and whether integrative encoding of melodies with new information can facilitate learning in normal subjects and subjects with memory impairments.

## Data availability statement

The datasets presented in this study can be found in online repositories. The names of the repository/repositories and accession number(s) can be found at: Open Science Framework: https://osf.io/63mep/.

## Ethics statement

The studies involving human participants were reviewed and approved by Ethics Committee of the Charité – Universitätsmedizin Berlin. The patients/participants provided their written informed consent to participate in this study.

## Author contributions

MH, AS, and CP designed and conceptualized the study, analyzed and interpreted the data, drafted, reviewed, and edited the manuscript. MH collected the data. All authors contributed to the article and approved the submitted version.

## Funding

MH and AS were supported by the Bundesministerium für Bildung und Forschung (01PL16032). CP was supported by the Deutsche Forschungsgemeinschaft (DFG, German Research Foundation) – Project number 327654276 – SFB 1315.

## Conflict of interest

The authors declare that the research was conducted in the absence of any commercial or financial relationships that could be construed as a potential conflict of interest.

## Publisher’s note

All claims expressed in this article are solely those of the authors and do not necessarily represent those of their affiliated organizations, or those of the publisher, the editors and the reviewers. Any product that may be evaluated in this article, or claim that may be made by its manufacturer, is not guaranteed or endorsed by the publisher.
