# Supplementary material for: Musical expertise shapes visual-melodic memory integration
Source: Front Psychol. 2022 Oct 24;13:973164. doi: 10.3389/fpsyg.2022.973164 (PMC9637918; doi:10.3389/fpsyg.2022.973164)
Supplement: Supplementary file 1 [file Table_1.docx]

Supplementary information

**Supplementary Table 1.** List of musical stimuli.

| **Composer** | **Title** | **Genre** | **Length** |
| --- | --- | --- | --- |
| Komarowski, Anatoli | Violin concerto | Classic | 00:07 |
| Badings, Henk | Scherzo Pastorale (from Little Piano Pieces) | Classic | 00:06 |
| Bartok, Bela | Bulgarian Rhythms - 4th mvt | Classic | 00:06 |
| Thielemans, Toots | Bluesette | Jazz | 00:07 |
| Bolling, Claude | Javanaise (from Suite for Flute and Jazz Piano) | Jazz/Classic | 00:06 |
| Bortkiewicz, Sergei E. | The Princess and the Pie (from Andersen's Fairy Tales) | Classic | 00:05 |
| Debussy, Claude | Passepied (from Suite Bergamasque) | Classic | 00:07 |
| Chabrier, Emmanuel | Espana | Classic | 00:08 |
| Gulda, Friedrich | Moderato (from Play Piano Play) | Jazz | 00:07 |
| Busch, Elliot | Ivory Rag | Jazz | 00:08 |
| Janáček, Leoš | No. 5 (from On an Overgrown Path) | Classic | 00:05 |
| Janáček, Leoš | Moderato (from Sinfonietta) | Classic | 00:07 |
| Khachaturian, Aram | Andantino (from Album for Children No. 1) | Classic | 00:10 |
| Manuel, Horacio | Balada Da Felicidade | - | 00:08 |
| Mays, Lyle | Chorinho | Jazz | 00:06 |
| Pachelbel, Johann | Magnificat fugue secundi toni No. 2 | Classic | 00:06 |
| Poulenc, Francis | Mouvements perpétuels | Classic | 00:06 |
| Prokofiev, Sergei | Vivace (from Piano Sonata No. 6) | Classic | 00:05 |
| Skrjabin, Alexander | No. 4 Lento (from 5 Préludes) | Classic | 00:06 |
| Portillo de la Luz, César | Son al Son | Traditional | 00:06 |
| Taki, Rentaro | Moon over ruined castle | Traditional/Folk song | 00:07 |
| Troup, Bobby | Their hearts were full of spring | Pop | 00:05 |
| Tchaikovsky, Pyotr I. | Chanson Triste op. 40/No. 2 | Classic | 00:06 |
| Joplin, Scott | Fig Leaf Rag | Jazz | 00:07 |
| Gounod, Charles | Funeral March of a Marionette | Classic | 00:08 |
| Moszkowski, Moritz | Spanish Dance No. 2 | Classic | 00:05 |
| Copland, Aaron | Three Moods, Jazzy | Jazz | 00:09 |
| O'Hagan, Jack | Road to Gundagai | Traditional/Folk song | 00:08 |
| Gershwin, George | Rialto Ripple Rag | Classic/Jazz | 00:07 |
| Ascher, Emil | Arabian Wedding March | Classic | 00:08 |
| Riedel, Georg | Idas Sommarvisa | Folk song | 00:09 |
| Bonnet, Carlos | La partida (Vals venezolana) | Traditional | 00:07 |
| - | Saka Naka | Traditional/Folk song | 00:08 |
| - | Klezmeron | Traditional | 00:08 |
| Johnson, Laurie | The Avengers | Theme Song/Film song | 00:07 |
| Stein, Herman | Gunsmoke | Theme Song/Film song | 00:08 |
| Vars, Henry | Flipper | Theme Song/Film song | 00:07 |
| Rose, David | Little House -A new beginning | Theme Song/Film song | 00:06 |
| Beethoven, Ludwig v. | Return to Ulster (from 25 Irish Songs) | Classic | 00:10 |
| Tugarinov, J. | Vo pole berjesa stojala | Traditional/Folk song | 00:10 |
| Anonymous/Thomas Oliphant | The Ash Grove | Traditional/Folk song | 00:07 |
| Copland, Aaron | Appalachian Suite | Classic | 00:08 |
| - | Vermeland | Traditional/Folks song | 00:07 |

**Supplementary Information and Supplementary Table 2.** Influence of melody length on accuracy in direct trials

Since the length of melodies varied considerably and might have influenced performance in the task, we calculated a logistic regression with melody length and group as predictors and accuracy in direct trials (i.e. correctly / incorrectly answered direct trials) as dependent variable. The results of this analysis are reported in Supplementary Table 2. The results showed that group significantly predicted whether a direct trial was answered correctly or not. However, melody length did not increase the odds of answering a trial correctly.

| Predictor | *β* (*SE*) | *z* value | *p* value | Odds ratio |
| --- | --- | --- | --- | --- |
| Intercept | 1.12 (0.24) | 4.59 | <0.001 |  |
| Melody Length | -0.01 (0.03) | -0.34 | 0.74 | 0.99 |
| Group | 0.65 (0.09) | 7.37 | <0.001 | 1.92 |

*Note.* Model χ^2^(2) = 55.91, *p* < 0.001; *SE* = standard error
